# Crystal structure of dilithium potassium citrate, Li_2_KC_6_H_5_O_7_ determined from powder diffraction data and DFT calculations

**DOI:** 10.1107/S2056989019002809

**Published:** 2019-02-28

**Authors:** Andrew J. Cigler, James A. Kaduk

**Affiliations:** a Department of Chemistry, North Central College, 131 S. Loomis St, Naperville IL, 60540 , USA

**Keywords:** powder diffraction, density functional theory, citrate, lithium, potassium

## Abstract

The crystal structure of poly[μ-citrato-dilithium(I)potassium(I)], [Li_2_K(C_6_H_5_O_7_)]_*n*_, has been solved and refined using laboratory X-ray powder diffraction data, and optimized using density functional techniques. The citrate anion triply chelates to the K^+^ cation through the hydroxyl group, the central carboxyl­ate, and the terminal carboxyl­ate. The KO_7_ coordination polyhedra share edges, forming chains parallel to the *a* axis. These chains share edges with one tetra­hedral Li ion, and are bridged by edge-sharing pairs of the second tetra­hedral Li ion, forming layers parallel to the *ac* plane.

## Abstract 2

The crystal structure of dilithium potassium citrate has been solved and refined using laboratory X-ray powder diffraction data, and optimized using density functional techniques. The KO_7_ coordination polyhedra share edges, forming chains parallel to the *a* axis. These chains share edges with one tetra­hedral Li, and are bridged by edge-sharing pairs of the second tetra­hedral Li, forming layers parallel to the *ac* plane.

## Chemical context   

A systematic study of the crystal structures of Group 1 (alkali metal) citrate salts has been reported in Rammohan & Kaduk (2018 ▸). The study was extended to lithium metal hydrogen citrates in Cigler & Kaduk (2018 ▸), to sodium metal hydrogen citrates in Cigler & Kaduk (2019*a* ▸), and to sodium dirubidium citrates in Cigler & Kaduk (2019*b* ▸). We now describe the synthesis and structure of the title compound, Li_2_KC_6_H_5_O_7_, which represents a further extension to the family of known lithium potassium citrates. Only one mixed lithium potassium citrate has been reported previously: the double salt LiK_2_(HC_6_H_5_O_7_)(H_2_CH_5_O_7_)(H_2_O) [CSD (Groom *et al.*, 2016 ▸) refcode LATPOL; Zacharias & Glusker, 1993 ▸].
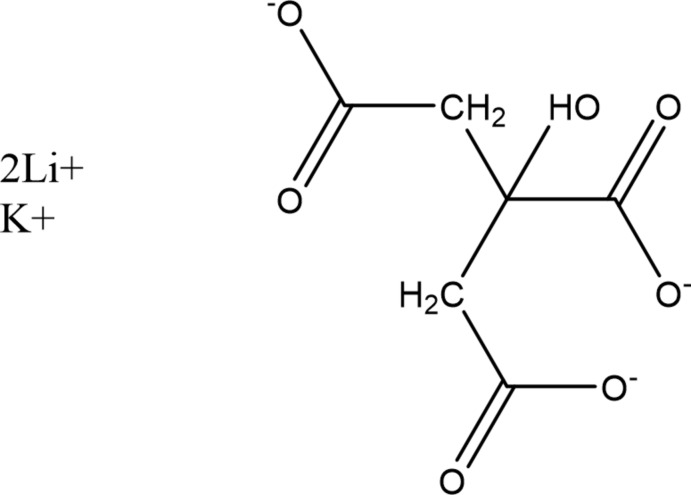


## Structural commentary   

**Figure 1:**
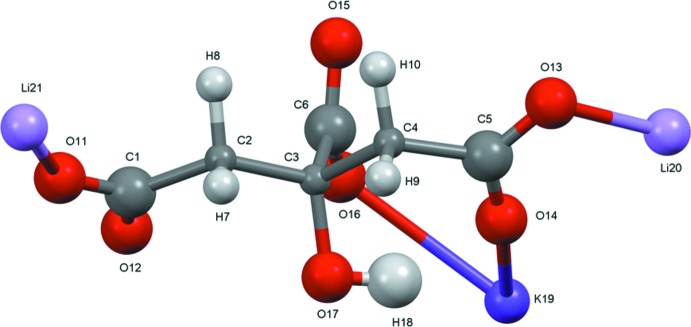
The asymmetric unit of Li_2_KC_6_H_5_O_7_ with the atom numbering and 50% probability spheres.

**Figure 2:**
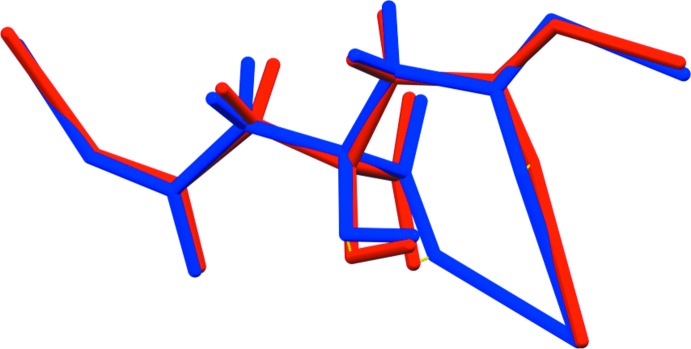
Comparison of the refined and optimized structures of Li_2_KC_6_H_5_O_7_. The refined structure is in red, and the DFT-optimized structure is in blue.

The structure of Li_2_KC_6_H_5_O_7_ was solved and refined from powder data and optimized by density functional theory (DFT) calculations (see *Experimental* Section) and is illustrated in Fig. 1 ▸. The root-mean-square Cartesian displacement of the non-hydrogen atoms in the refined and optimized structures is 0.24 Å (Fig. 2 ▸). The largest differences (0.3–0.4 Å) are in the K19 coordination sphere. The general good agreement between the structures is evidence that the experimental structure is correct (van de Streek & Neumann, 2014 ▸). All of the citrate bond distances, bond angles, and torsion angles fall within the normal ranges indicated by a *Mercury* Mogul Geometry Check (Macrae *et al.*, 2008 ▸). The citrate anion occurs in the *trans,trans*-conformation (about C2—C3 and C3—C4), which is one of the two low-energy conformations of an isolated citrate (Rammohan & Kaduk, 2018 ▸). The central carboxyl­ate group and the hy­droxy group exhibit a small twist [O16—C6—C3—O17 torsion angle 7.7°] from the normal planar arrangement. The Mulliken overlap populations indicate that both the Li—O and K—O bonds have some covalent character, but that the Li—O bonds are more covalent.

The citrate anion triply chelates to K19 though the hydroxyl group O17, the central carboxyl­ate group (atom O16), and the terminal carboxyl­ate (O12). Each citrate oxygen atom (except O14, which only bonds to K19) bridges multiple metal atoms. K19 is seven-coordinate (irregular), with a bond-valence sum of 1.15. Li20 and Li21 are tetra­hedral, with bond-valence sums of 0.95 and 1.08, respectively.

The Bravais–Friedel–Donnay–Harker (Bravais, 1866 ▸; Friedel, 1907 ▸; Donnay & Harker, 1937 ▸) method suggests that we might expect platy morphology for dilithium potassium citrate, with {001} as the principal faces. A 2nd order spherical harmonic preferred orientation model was included in the refinement; the texture index was 1.012, indicating that preferred orientation was very slight for this rotated capillary specimen.

## Supra­molecular features   

**Figure 3:**
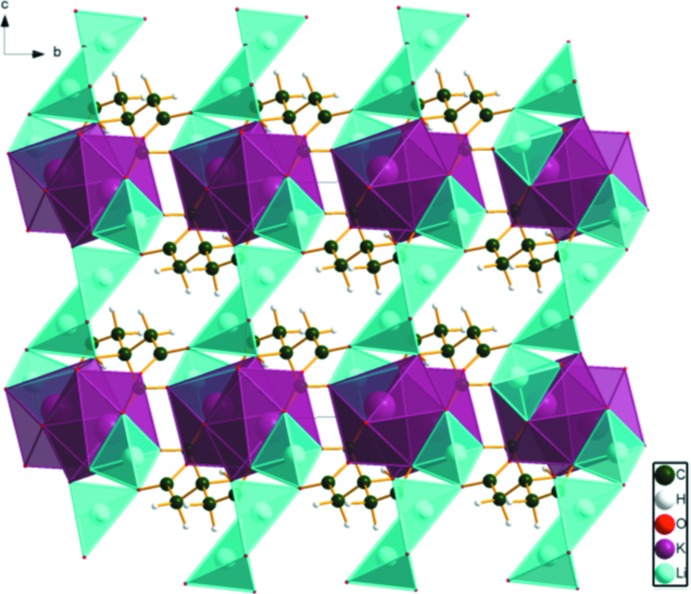
Crystal structure of Li_2_KC_6_H_5_O_7_, viewed down the *a*-axis direction.

**Figure 4:**
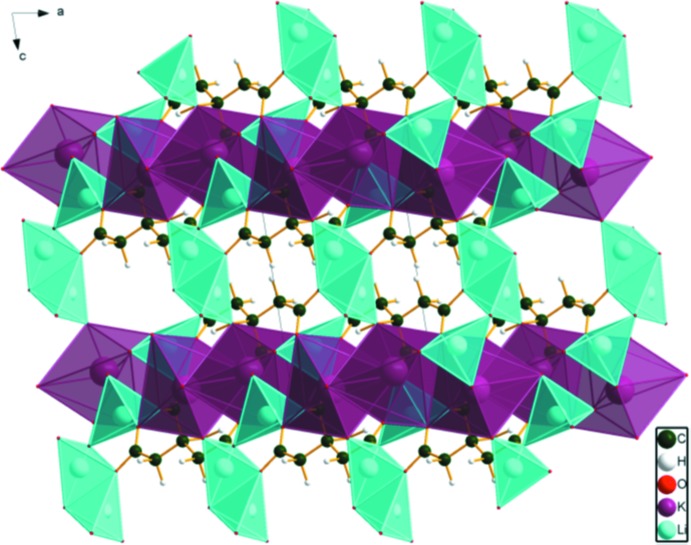
Crystal structure of Li_2_KC_6_H_5_O_7_, viewed down the *b*-axis direction.

The KO_7_ coordination polyhedra share edges, forming chains parallel to the *a*-axis direction (Fig. 3 ▸). These chains share edges with Li20, and are bridged by edge-sharing pairs of Li21, forming layers lying parallel to the *ac* plane (Figs. 4 ▸ and 5 ▸).

The only traditional hydrogen bond is an intra­molecular O17—H18⋯O11 link between the hydroxyl group and one of the terminal carboxyl­ate groups (Table 1 ▸). By the correlation of Rammohan & Kaduk (2018 ▸), this hydrogen bond contributes about 13.9 kcal mol^−1^ to the crystal energy. There is also a weak intra­molecular C—H⋯O hydrogen bond (Table 1 ▸).

## Database survey   

Details of the comprehensive literature search for citrate structures are presented in Rammohan & Kaduk (2018 ▸). The pattern of Li_2_KC_6_H_5_O_7_ was indexed using *N-TREOR* (Altomare *et al.*, 2013 ▸), and the cell was reduced using the tools in the PDF-4+ database (Fawcett *et al.*, 2017 ▸). A reduced cell search in the Cambridge Structural Database (Groom *et al.*, 2016 ▸) yielded no hits.

## Synthesis and crystallization   

0.7412 g Li_2_CO_3_ (10.0 mmol, Sigma–Aldrich) and 0.6910 g K_2_CO_3_ (5.0 mmol, Sigma–Aldrich) were added to a solution of 2.0175 g citric acid (10.0 mmol, Sigma–Aldrich) monohydrate in 10 ml water. After the fizzing subsided, the clear solution was dried at 338 K to yield a clear glass. The glass was heated at 423 K for 30 min to yield a slightly hygroscopic white solid.

## Refinement   

**Figure 6:**
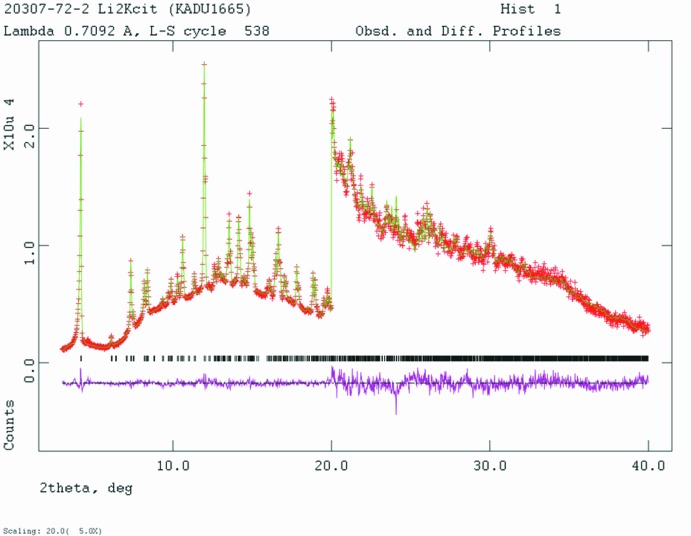
Rietveld plot for Li_2_KC_6_H_5_O_7_. The red crosses represent the observed data points, and the green line is the calculated pattern. The magenta curve is the difference pattern, plotted at the same scale as the other patterns. The vertical scale has been multiplied by a factor of five for 2θ > 20.0°. The row of black tick marks indicates the reflection positions for this phase.

Crystal data, data collection and structure refinement details are summarized in Table 2 ▸. A Rietveld plot is shown in Fig. 6 ▸. The structure of Li_2_KC_6_H_5_O_7_ was solved using Monte Carlo-simulated annealing techniques as implemented in *EXPO2014* (Altomare *et al.*, 2013 ▸). A citrate anion, a K cation, and two Li cations were used as fragments. The positions of H7–H10 were calculated using *Materials Studio* (Dassault, 2018 ▸). The position of the active (ionizable) hydrogen atom H18 was deduced from the potential intra­molecular hydrogen-bonding pattern. The Li positions were unreasonable, so they were deleted from the model. Potential Li positions were identified by using *Materials Studio* to search for voids in the structure, with a Connelly radius of 0.9 Å. Pseudo-Voigt profile coefficients were as parameterized in Thompson *et al.* (1987 ▸) and the asymmetry correction of Finger *et al.* (1994 ▸) was applied and microstrain broadening by Stephens (1999 ▸). The hydrogen atoms were included in fixed positions, which were re-calculated during the course of the refinement using *Materials Studio*. The *U*_iso_ values of C2, C3, and C4 were constrained to be equal, and those of H7, H8, H9, and H10 were constrained to be 1.3× that of these carbon atoms. The *U*_iso_ values for C1, C5, C6, and the oxygen atoms were constrained to be equal, and that of H18 was constrained to be 1.3× this value. The *U*_iso_ values of Li20 and Li21 were fixed. An 11-term diffuse scattering function was used to describe the scattering from the capillary and the significant fraction of amorphous material.

A density functional geometry optimization (fixed experimental unit cell) was carried out using *CRYSTAL14* (Dovesi *et al.*, 2014 ▸). The basis sets for the H, C, and O atoms were those of Gatti *et al.* (1994 ▸), and the basis sets for Li and K were those of Peintinger *et al.* (2013 ▸). The calculation was run on eight 2.1 GHz Xeon cores (each with 6 GB RAM) of a 304-core Dell Linux cluster at Illinois Institute of Technology, using 8 *k*-points and the B3LYP functional, and took 18 h.

## Supplementary Material

Crystal structure: contains datablock(s) KADU1665_publ, KADU1665_DFT. DOI: 10.1107/S2056989019002809/hb7800sup1.cif

Supporting information file. DOI: 10.1107/S2056989019002809/hb7800KADU1665_publsup2.cml

CCDC references: 1899380, 1899381

Additional supporting information:  crystallographic information; 3D view; checkCIF report

## Figures and Tables

**Figure 5:**
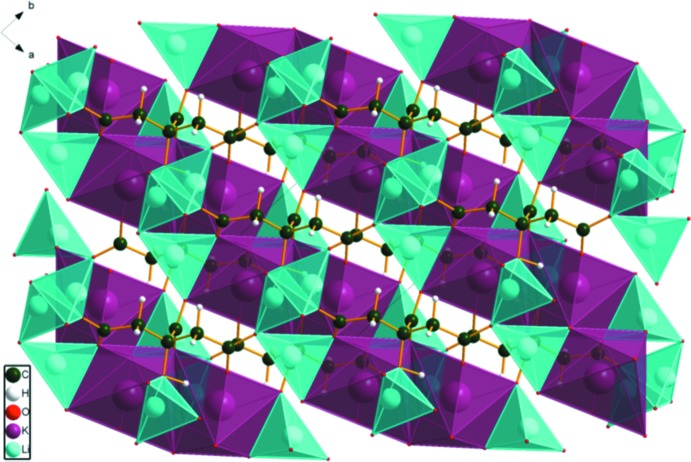
Crystal structure of Li_2_KC_6_H_5_O_7_, viewed down the *c*-axis direction.

**Table 1:** Hydrogen-bond geometry (Å, °, electrons, kcal mol^−1^) for [Li_2_K(C_6_H_5_O_7_)]

*D*—H⋯*A*′	*D*—H	H⋯*A*	*D*⋯*A*	*D*—H⋯*A*	Mulliken overlap	H-bond energy
O17—H18⋯O14	0.987	1.786	2.662	145.8	0.065	13.9
C4—H10⋯O15	1.090	2.516	2.770	91.6	0.010	

**Table 2:** Experimental details

Crystal data	
Chemical formula	[Li_2_K(C_6_H_5_O_7_)]
*M*_r_	242.08
Crystal system, space group	Triclinic, *P* 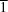
Temperature (K)	302
*a*, *b*, *c* (Å)	6.4842 (3), 6.6833 (3), 9.8171 (4)
α, β, γ (°)	87.637 (4), 80.606 (4), 83.109 (4)
*V* (Å^3^)	416.59 (2)
*Z*	2
Radiation type	*K*α_1_, *K*α_2_, λ = 0.709237, 0.713647 Å
Specimen shape, size (mm)	Cylinder, 12 × 1

Data collection	
Diffractometer	PANalytical Empyrean
Specimen mounting	Glass capillary
Data collection mode	Transmission
Scan method	Step
2θ values (°)	2θ_min_ = 1.008, 2θ_max_ = 49.988, 2θ_step_ = 0.017

Refinement	
*R* factors and goodness of fit	*R*_p_ = 0.016, *R*_wp_ = 0.021, *R*_exp_ = 0.013, *R*(*F* ^2^) = 0.13685, χ^2^ = 2.722
No. of parameters	79
No. of restraints	29
H-atom treatment	Only H-atom displacement parameters refined

The same symmetry and lattice parameters were used for the DFT calculations as for the powder diffraction study. Computer programs: *Data Collector* (PANalytical, 2011 ▸), *PowDLL* (Kourkoumelis, 2013 ▸), *EXPO2014* (Altomare *et al.*, 2013 ▸), *GSAS* (Larson & Von Dreele, 2004 ▸), *Mercury* (Macrae *et al.*, 2008 ▸), *DIAMOND* (Putz & Brandenburg, 2015 ▸) and *publCIF* (Westrip, 2010 ▸).

## supplementary crystallographic information

### dilithium potassium citrate (KADU1665_publ) Crystal data

**Table:**

[Li_2_K(C_6_H_5_O_7_)]	γ = 83.109 (4)°
*M**_r_* = 242.08	*V* = 416.59 (2) Å^3^
Triclinic, *P*1	*Z* = 2
Hall symbol: -P 1	*D*_x_ = 1.930 Mg m^−^^3^
*a* = 6.4842 (3) Å	Kα_1_, Kα_2_ radiation, λ = 0.709237, 0.713647 Å
*b* = 6.6833 (3) Å	*T* = 302 K
*c* = 9.8171 (4) Å	white
α = 87.637 (4)°	cylinder, 12 × 1 mm
β = 80.606 (4)°	Specimen preparation: Prepared at 423 K

### dilithium potassium citrate (KADU1665_publ) Data collection

**Table:**

PANalytical Empyrean diffractometer	Data collection mode: transmission
Radiation source: sealed X-ray tube	Scan method: step
Specimen mounting: glass capillary	2θ_min_ = 1.008°, 2θ_max_ = 49.988°, 2θ_step_ = 0.017°

### dilithium potassium citrate (KADU1665_publ) Refinement

**Table:**

Least-squares matrix: full	79 parameters
*R*_p_ = 0.016	29 restraints
*R*_wp_ = 0.021	3 constraints
*R*_exp_ = 0.013	Only H-atom displacement parameters refined
*R*(*F*^2^) = 0.13685	Weighting scheme based on measured s.u.'s
2932 data points	(Δ/σ)_max_ = 0.06
Excluded region(s): 1-3 degrees. The asymmetry of the low-angle peak made it hard to model.	Background function: GSAS Background function number 1 with 1 terms. Shifted Chebyshev function of 1st kind 1: 2122.50
Profile function: CW Profile function number 3 with 19 terms Pseudovoigt profile coefficients as parameterized in Thompson *et al.* (1987). Asymmetry correction of Finger *et al.* (1994).	

### dilithium potassium citrate (KADU1665_publ) Special details

**Table:**

Refinement. VERSION 6 DESCR 20307-72-2 Li2Kcit (KADU1665) HSTRY 1 created readexp.tcl 1251 2016-07-26T14:01:41 HSTRY 2 EXPTOOL Win32 Jul 26 14:02:04 2016 P P HSTRY 3 EXPTOOL Win32 Jul 26 14:02:07 2016 P A HSTRY 4 EXPGUI 1251 1251 (27 changes) – 07/26/16 14:03:39 HSTRY 5 EXPTOOL Win32 Jul 26 14:03:39 2016 P H HSTRY 6 EXPGUI 1251 1251 (36 changes) – 07/26/16 14:04:24 HSTRY 7 POWPREF Win32 Jul 26 14:04:24 2016 HSTRY 8 GENLES Win32 Jul 26 14:04:28 2016 Sdsq= 0.809E+06 S/E= 0.356E-02 HSTRY 9 EXPGUI 1251 1251 (5 changes) – 07/26/16 14:05:27 HSTRY 10 POWPREF Win32 Jul 26 14:05:27 2016 HSTRY 11 GENLES Win32 Jul 26 14:05:29 2016 Sdsq= 0.840E+06 S/E= 0.822E-08 HSTRY 12 EXPEDT Win32 Jul 26 14:35:31 2016 L O HSTRY 13 POWPREF Win32 Jul 26 14:35:39 2016 HSTRY 14 GENLES Win32 Jul 26 14:35:46 2016 Sdsq= 0.458E+06 S/E= 82.7 HSTRY 15 EXPGUI 1251 1251 (17 changes) – 07/26/16 14:37:28 HSTRY 16 POWPREF Win32 Jul 26 14:37:28 2016 HSTRY 17 GENLES Win32 Jul 26 14:37:32 2016 Sdsq= 0.411E+06 S/E= 8.80 HSTRY 18 EXPGUI 1251 1251 (6 changes) – 07/26/16 14:40:01 HSTRY 19 EXPEDT Win32 Jul 26 14:46:39 2016 L S A HSTRY 20 POWPREF Win32 Jul 26 14:47:02 2016 HSTRY 21 GENLES Win32 Jul 26 14:47:11 2016 Sdsq= 0.301E+06 S/E= 163. HSTRY 22 EXPGUI 1251 1251 (3 changes) – 07/26/16 14:47:33 HSTRY 23 GENLES Win32 Jul 26 14:47:56 2016 Sdsq= 0.241E+06 S/E= 20.1 HSTRY 24 GENLES Win32 Jul 26 14:48:26 2016 Sdsq= 0.241E+06 S/E= 0.233 HSTRY 25 EXPGUI 1251 1251 (3 changes) – 07/26/16 14:49:54 HSTRY 26 EXPGUI 1251 1251 (2 changes) – 07/26/16 14:52:55 HSTRY 27 GENLES Win32 Jul 26 14:53:00 2016 Sdsq= 0.302E+06 S/E= 20.3 HSTRY 28 EXPGUI 1251 1251 (3 changes) – 07/26/16 15:02:46 HSTRY 29 GENLES Win32 Jul 26 15:02:51 2016 Sdsq= 0.280E+06 S/E= 47.5 HSTRY 30 EXPGUI 1251 1251 (1 changes) – 07/26/16 15:08:11 HSTRY 31 EXPGUI 1251 1251 (20 changes) – 07/26/16 18:07:53 HSTRY 32 EXPTOOL Win32 Jul 26 18:07:53 2016 P A HSTRY 33 EXPEDT Win32 Jul 26 18:08:05 2016 L S A HSTRY 34 POWPREF Win32 Jul 26 18:08:11 2016 HSTRY 35 GENLES Win32 Jul 26 18:08:30 2016 Sdsq= 0.383E+06 S/E= 86.5 HSTRY 36 EXPGUI 1251 1251 (6 changes) – 07/26/16 18:09:17 HSTRY 37 GENLES Win32 Jul 26 18:09:35 2016 Sdsq= 0.194E+06 S/E= 80.0 HSTRY 38 GENLES Win32 Jul 26 18:10:02 2016 Sdsq= 0.184E+06 S/E= 9.15 HSTRY 39 EXPEDT Win32 Jul 26 18:16:32 2016 F-EDT HSTRY 40 EXPEDT Win32 Jul 26 18:24:22 2016 LA HSTRY 41 GENLES Win32 Jul 26 18:24:42 2016 Sdsq= 0.188E+06 S/E= 27.3 HSTRY 42 EXPEDT Win32 Jul 26 18:25:46 2016 LA HSTRY 43 GENLES Win32 Jul 26 18:26:06 2016 Sdsq= 0.180E+06 S/E= 33.5 HSTRY 44 EXPEDT Win32 Jul 26 18:27:34 2016 L O HSTRY 45 GENLES Win32 Jul 26 18:27:55 2016 Sdsq= 0.133E+06 S/E= 87.6 HSTRY 46 GENLES Win32 Jul 26 18:29:04 2016 Sdsq= 0.128E+06 S/E= 26.1 HSTRY 47 GENLES Win32 Jul 26 18:30:18 2016 Sdsq= 0.126E+06 S/E= 27.7 HSTRY 48 EXPEDT Win32 Jul 26 18:30:56 2016 LA HSTRY 49 EXPEDT Win32 Jul 27 07:25:21 2016 P H LA O HSTRY 50 POWPREF Win32 Jul 27 07:25:24 2016 HSTRY 51 GENLES Win32 Jul 27 07:25:33 2016 Sdsq= 0.126E+06 S/E= 171. HSTRY 52 POWPREF Win32 Jul 27 07:25:52 2016 HSTRY 53 GENLES Win32 Jul 27 07:26:01 2016 Sdsq= 0.116E+06 S/E= 3.33 HSTRY 54 EXPEDT Win32 Jul 27 07:27:18 2016 L B O HSTRY 55 GENLES Win32 Jul 27 07:27:22 2016 Sdsq= 0.906E+06 S/E= 0.285E-08 HSTRY 56 EXPEDT Win32 Jul 27 07:29:10 2016 L O HSTRY 57 GENLES Win32 Jul 27 07:29:19 2016 Sdsq= 0.194E+06 S/E= 430. HSTRY 58 GENLES Win32 Jul 27 07:29:30 2016 Sdsq= 0.165E+06 S/E= 9.08 HSTRY 59 EXPEDT Win32 Jul 27 07:30:47 2016 L O HSTRY 60 GENLES Win32 Jul 27 07:30:56 2016 Sdsq= 0.805E+05 S/E= 53.8 HSTRY 61 GENLES Win32 Jul 27 07:31:07 2016 Sdsq= 0.452E+05 S/E= 2.30 HSTRY 62 EXPEDT Win32 Jul 27 07:31:40 2016 L O HSTRY 63 POWPREF Win32 Jul 27 07:31:43 2016 HSTRY 64 GENLES Win32 Jul 27 07:31:53 2016 Sdsq= 0.266E+05 S/E= 27.7 HSTRY 65 POWPREF Win32 Jul 27 07:32:16 2016 HSTRY 66 GENLES Win32 Jul 27 07:32:25 2016 Sdsq= 0.261E+05 S/E= 35.7 HSTRY 67 EXPEDT Win32 Jul 27 07:33:18 2016 L O HSTRY 68 GENLES Win32 Jul 27 07:33:26 2016 Sdsq= 0.261E+05 S/E= 46.5 HSTRY 69 EXPEDT Win32 Jul 27 07:33:53 2016 L O HSTRY 70 GENLES Win32 Jul 27 07:34:02 2016 Sdsq= 0.263E+05 S/E= 9.83 HSTRY 71 EXPEDT Win32 Jul 27 07:34:15 2016 L O HSTRY 72 GENLES Win32 Jul 27 07:34:21 2016 Sdsq= 0.261E+05 S/E= 0.351E-02 HSTRY 73 EXPEDT Win32 Jul 27 07:34:59 2016 LA HSTRY 74 POWPREF Win32 Jul 27 07:35:02 2016 HSTRY 75 GENLES Win32 Jul 27 07:35:11 2016 Sdsq= 0.158E+05 S/E= 69.8 HSTRY 76 EXPEDT Win32 Jul 27 07:35:33 2016 LA HSTRY 77 GENLES Win32 Jul 27 07:35:42 2016 Sdsq= 0.145E+05 S/E= 22.3 HSTRY 78 GENLES Win32 Jul 27 07:35:52 2016 Sdsq= 0.142E+05 S/E= 3.90 HSTRY 79 EXPTOOL Win32 Jul 28 08:41:14 2016 P A HSTRY 80 EXPGUI 1251 1251 (3 changes) – 07/28/16 08:43:24 HSTRY 81 GENLES Win32 Jul 28 08:44:01 2016 Sdsq= 0.130E+05 S/E= 77.6 HSTRY 82 GENLES Win32 Jul 28 08:44:17 2016 Sdsq= 0.122E+05 S/E= 5.81 HSTRY 83 EXPGUI 1251 1251 (86 changes) – 07/28/16 08:54:30 HSTRY 84 GENLES Win32 Jul 28 08:54:36 2016 Sdsq= 0.122E+05 S/E= 1.39 HSTRY 85 EXPGUI 1251 1251 (3 changes) – 07/28/16 08:57:04 HSTRY 86 GENLES Win32 Jul 28 08:57:10 2016 Sdsq= 0.121E+05 S/E= 1.37 HSTRY 87 EXPGUI 1251 1251 (3 changes) – 07/28/16 08:58:42 HSTRY 88 GENLES Win32 Jul 28 08:58:48 2016 Sdsq= 0.122E+05 S/E= 0.813E-01 HSTRY 89 EXPGUI 1251 1251 (16 changes) – 07/28/16 09:04:01 HSTRY 90 GENLES Win32 Jul 28 09:04:07 2016 Sdsq= 0.109E+05 S/E= 181. HSTRY 91 EXPGUI 1251 1251 (24 changes) – 07/28/16 09:08:38 HSTRY 92 POWPREF Win32 Jul 28 09:08:38 2016 HSTRY 93 GENLES Win32 Jul 28 09:08:44 2016 Sdsq= 0.110E+05 S/E= 25.6 HSTRY 94 EXPGUI 1251 1251 (1 changes) – 07/28/16 09:09:55 HSTRY 95 POWPREF Win32 Jul 28 09:09:55 2016 HSTRY 96 GENLES Win32 Jul 28 09:10:00 2016 Sdsq= 0.106E+05 S/E= 21.4 HSTRY 97 EXPGUI 1251 1251 (2 changes) – 07/28/16 09:23:11 HSTRY 98 GENLES Win32 Jul 28 09:23:14 2016 Sdsq= 0.105E+05 S/E= 3.06 HSTRY 99 EXPGUI 1251 1251 (9 changes) – 07/28/16 09:23:53 HSTRY100 POWPREF Win32 Jul 28 09:23:56 2016 HSTRY101 GENLES Win32 Jul 28 09:24:00 2016 Sdsq= 0.107E+05 S/E= 16.0 HSTRY102 GENLES Win32 Jul 28 09:24:10 2016 Sdsq= 0.106E+05 S/E= 2.42 HSTRY103 GENLES Win32 Jul 28 09:24:21 2016 Sdsq= 0.105E+05 S/E= 0.422 HSTRY104 EXPGUI 1251 1251 (1 changes) – 07/28/16 09:25:54 HSTRY105 GENLES Win32 Jul 28 09:25:57 2016 Sdsq= 0.105E+05 S/E= 0.775E-01 HSTRY106 EXPEDT Win32 Jul 28 09:26:34 2016 L O HSTRY107 GENLES Win32 Jul 28 09:26:44 2016 Sdsq= 0.940E+04 S/E= 51.7 HSTRY108 GENLES Win32 Jul 28 09:26:54 2016 Sdsq= 0.890E+04 S/E= 7.94 HSTRY109 EXPEDT Win32 Jul 28 09:29:43 2016 L O HSTRY110 GENLES Win32 Jul 28 09:29:53 2016 Sdsq= 0.644E+04 S/E= 179. HSTRY111 GENLES Win32 Jul 28 09:30:05 2016 Sdsq= 0.592E+04 S/E= 30.2 HSTRY112 GENLES Win32 Jul 28 09:30:15 2016 Sdsq= 0.583E+04 S/E= 4.76 HSTRY113 EXPGUI 1251 1251 (1 changes) – 07/28/16 09:31:21 HSTRY114 EXPGUI 1251 1251 (3 changes) – 07/28/16 09:32:17 HSTRY115 POWPREF Win32 Jul 28 09:32:17 2016 HSTRY116 GENLES Win32 Jul 28 09:32:23 2016 Sdsq= 0.594E+04 S/E= 5.43 HSTRY117 GENLES Win32 Jul 28 09:32:31 2016 Sdsq= 0.592E+04 S/E= 0.788 HSTRY118 GENLES Win32 Jul 28 09:32:38 2016 Sdsq= 0.591E+04 S/E= 0.115 HSTRY119 GENLES Win32 Jul 28 09:33:35 2016 Sdsq= 0.591E+04 S/E= 0.171E-01 HSTRY120 GENLES Win32 Jul 28 09:33:43 2016 Sdsq= 0.591E+04 S/E= 0.923E-02 HSTRY121 EXPGUI 1251 1251 (1 changes) – 07/28/16 09:34:25 HSTRY122 GENLES Win32 Jan 12 10:22:51 2019 Sdsq= 0.591E+04 S/E= 0.765E-02 HSTRY123 EXPGUI 1251 1251 (1 changes) – 01/12/19 10:23:21 DSGL CDAT1 DRAD ARAD NOFO FOUR CDAT1 DELF Z 1 NOPR 0.00 999.99 GNLS RUN on Jan 12 10:22:51 2019 Total cycles run 539 5912.1 GNLS CDAT1 MXCY 9MARQ1.00CVRG-200PRNT 258 GNLS SHFTS 0.06 0.00 AFAC C 2.310020.8439 1.020010.2075 1.5886 .5687 .865051.6512 .2156 RHF AFAC C_ 12.011 .6646 .0035 AFAC C_SIZ 1.12 0.92 1.60 3 AFAC C_XAB 288.3 170.4 84.13 10.67 7.054 52.30 141.0 AFAC C_XF1 0.036 0.027 0.018 0.003 0.002 0.049 0.024-0.001-0.001-0.001 AFAC C_XF2 0.021 0.015 0.009 0.002 0.001 0.031 0.012 0.000 0.000 0.000 AFAC H .49300210.5109.32291226.1257.1401913.14236.04081057.7997.003038 SDS AFAC H_ 1.008-.3739 20.6 19.2 AFAC H_SIZ 0.98 0.78 1.20 118 AFAC H_XAB .6888 .6700 .6548 .6238 .6131 .7240 .6640 AFAC H_XF1 0. 0. 0. 0. 0. 0. 0. 0. 0. 0. AFAC H_XF2 0. 0. 0. 0. 0. 0. 0. 0. 0. 0. AFAC K 8.218612.7949 7.4398 .7748 1.0519213.187 .865941.6841 1.4228 RHF AFAC K_ 39.098 .367 2.1 AFAC K_SIZ 2.58 2.38 2.00 708 AFAC K_XAB 27710 17820 9637 1052 524.6 44100 14100 AFAC K_XF1 0.091 0.307 0.387 0.201 0.140-0.508 0.353 0.009 0.007-0.002 AFAC K_XF2 2.109 1.589 1.066 0.249 0.156 2.844 1.386 0.022 0.020 0.015 AFAC O 3.048513.2771 2.2868 5.7011 1.5463 .3239 .867032.9089 .2508 RHF AFAC O_ 15.999 .5803 .00019 AFAC O_SIZ 1.09 0.89 1.40 96 1.40 AFAC O_XAB 988.0 590.6 293.1 30.48 17.21 1730. 478.0 AFAC O_XF1 0.093 0.072 0.049 0.011 0.006 0.121 0.063-0.002-0.003-0.003 AFAC O_XF2 0.073 0.052 0.032 0.006 0.004 0.106 0.044 0.000 0.000 0.000 AFAC LI 1.1282 3.9546 .7508 1.0524 .617585.3905 .4653168.261 .0377 RHF AFAC LI_ 6.941 -.190 70.5 AFAC LI_SIZ 1.76 1.56 1.60 91 AFAC LI_XAB 14.32 9.193 5.496 2.268 2.061 2.510 7.990 AFAC LI_XF1 0.002 0.002 0.001 0.000 0.000 0.004 0.001-0.001-0.001-0.001 AFAC LI_XF2 0.001 0.001 0.000 0.000 0.000 0.001 0.000 0.000 0.000 0.000 AFAC SI 6.2915 2.4386 3.035332.3337 1.9891 .6785 1.541081.6937 1.1407 RHF AFAC SI_ 28.086.41491 .171 AFAC SI_SIZ 1.52 1.32 2.00 112 AFAC SI_XAB 9456 5878 3047 304.7 151.8 15200 4560. AFAC SI_XF1 0.365 0.321 0.254 0.082 0.052 0.392 0.298-0.002-0.002-0.006 AFAC SI_XF2 0.692 0.508 0.330 0.070 0.043 0.962 0.438 0.006 0.005 0.004 EXPR HTYP1 PXC RSN ANG EXPR NATYP 6 EXPR NHST 3 EXPR ATYP 1 C 6 1.129.200E-001 1.60 3 EXPR ATYP 2 H 59.800E-0017.800E-001 1.20 118 EXPR ATYP 3 O 7 1.098.900E-001 1.40 96 1.40 EXPR ATYP 4 K 1 2.58 2.38 2.00 708 EXPR ATYP 5 LI 2 1.76 1.56 1.60 91 EXPR ATYP 6 SI 0 1.52 1.32 2.00 112 EXPR DELFRL 443 EXPR NPHAS 1 0 0 0 0 0 0 0 0 REFN GDNFT Reduced CHI**2 = 2.732 for 79 variables REFN RESTR 29 297.17 REFN RPOWD 0.0214 0.0162 2214 5614.9 0.0000 0.0000 0 REFN STATS Cycle 539 There were 2243 observations. Total CHI**2 = 5.9121E+03 CIF AUTHOR James A. Kaduk CRS1 PNAM Li2 K C6 H5 O7 CRS1 NATOM 21 CRS1 ABC 6.484155 6.683338 9.817086 Y 0 CRS1 ABCSIG 0.000297 0.000340 0.000436 CRS1 ANGLES 87.6373 80.6064 83.1095 CRS1 ANGSIG 0.0041 0.0040 0.0039 CRS1 AT 1A C -0.155311 0.066381 0.326849 1.000000C1 2 086 CRS1 AT 1B 0.059833 I XU CRS1 AT 2A C -0.028510 0.232208 0.352910 1.000000C2 2 086 CRS1 AT 2B 0.022754 I XU CRS1 AT 3A C 0.186901 0.251260 0.263272 1.000000C3 2 086 CRS1 AT 3B 0.022754 I XU CRS1 AT 4A C 0.246282 0.429334 0.337748 1.000000C4 2 086 CRS1 AT 4B 0.022754 I XU CRS1 AT 5A C 0.434697 0.528184 0.268165 1.000000C5 2 086 CRS1 AT 5B 0.059833 I XU CRS1 AT 6A C 0.117993 0.348334 0.129209 1.000000C6 2 086 CRS1 AT 6B 0.059833 I XU CRS1 AT 7A H -0.054790 0.257310 0.443640 1.000000H7 2 006 CRS1 AT 7B 0.020581 I U CRS1 AT 8A H -0.111270 0.370550 0.282510 1.000000H8 2 006 CRS1 AT 8B 0.029581 I U CRS1 AT 9A H 0.371080 0.327240 0.432430 1.000000H9 2 006 CRS1 AT 9B 0.029581 I U CRS1 AT 10A H 0.162380 0.530640 0.404030 1.000000H10 2 006 CRS1 AT 10B 0.029581 I U CRS1 AT 11A O -0.309292 0.024533 0.417396 1.000000O11 2 086 CRS1 AT 11B 0.059833 I XU CRS1 AT 12A O -0.107542 -0.033386 0.216218 1.000000O12 2 086 CRS1 AT 12B 0.059833 I XU CRS1 AT 13A O 0.535822 0.667060 0.298831 1.000000O13 2 086 CRS1 AT 13B 0.059833 I XU CRS1 AT 14A O 0.547418 0.430562 0.169091 1.000000O14 2 086 CRS1 AT 14B 0.059833 I XU CRS1 AT 15A O 0.006116 0.520159 0.140518 1.000000O15 2 086 CRS1 AT 15B 0.059833 I XU CRS1 AT 16A O 0.208298 0.214588 0.041698 1.000000O16 2 086 CRS1 AT 16B 0.059833 I XU CRS1 AT 17A O 0.326946 0.072814 0.225143 1.000000O17 2 086 CRS1 AT 17B 0.059833 I XU CRS1 AT 18A H 0.394000 0.178500 0.233100 1.000000H18 2 006 CRS1 AT 18B 0.068781 I U CRS1 AT 19A K 0.740516 0.186092 -0.029707 1.000000K19 2 086 CRS1 AT 19B 0.046051 I XU CRS1 AT 20A LI 0.743494 0.745846 0.162760 1.000000Li20 2 086 CRS1 AT 20B 0.050000 I X CRS1 AT 21A LI 0.551237 0.101136 0.637623 1.000000Li21 2 086 CRS1 AT 21B 0.050000 I X CRS1 CELVOL 416.593 0.021 CRS1 CHMF 1 C 6.00 CRS1 CHMF 2 H 5.00 CRS1 CHMF 3 O 7.00 CRS1 CHMF 4 K 1.00 CRS1 CHMF 5 LI 2.00 CRS1 FMHST1 1 CRS1 FMPCTL 32 32 48 -2 -2 -3 18 18 51 CRS1 OD 2 5 0 NNNN 0 5 0.0000 0.0000 0.0000 CRS1 OD 1A 2 0 -2 2 0 -1 2 0 0 2 0 1 2 0 2 CRS1 OD 1B -0.1208 0.0453 -0.1888 0.0770 0.0181 CRS1 OD 1C0.2023E-010.1156E-010.2357E-010.1859E-010.1515E-01 CRS1 SG SYM P -1 CRS1 SPAXIS 0 0 1 HAP1 1 ZONE 0 0 0 0 HAP1 1EXTPOW 0.00000E+00 N 0 HAP1 1MASSFR 1.0000 0.0000 HAP1 1NAXIS 1 HAP1 1PHSFR 84.168 Y 0 HAP1 1PRCF 3 19 0.01000 9YNNNNNNNNNNNNNNNNNNYYYYYYYYNNNNNNNNN HAP1 1PRCF 1 0.215917E+03 0.000000E+00 0.900000E-02 0.000000E+00 HAP1 1PRCF 2 0.274800E+01 0.000000E+00 0.145554E-01 0.160000E-01 HAP1 1PRCF 3 0.000000E+00 0.000000E+00 0.000000E+00 0.000000E+00 HAP1 1PRCF 4 0.000000E+00 0.000000E+00 0.000000E+00 0.000000E+00 HAP1 1PRCF 5 0.000000E+00 0.000000E+00 0.000000E+00 HAP1 1PRCF 6 -0.221042E+00 0.382952E-01 -0.444951E-01 -0.554708E-01 HAP1 1PRCF 7 -0.235061E+00 -0.298567E+00 0.480744E+00 HAP1 1PREFO1 1.000000 1.000000 0.000000 0.000000 1.000000 NN 0 0 HAP1 1RADDAM 0.00000E+00 N 0 0.00 HST 1 HFIL kadu1665.gsas HST 1 HNAM 20307-72-2 Li2Kcit (60,40,1/4,0.02,1 mm cap) JAK HST 1 IFIL c:ζjak01νccpan.prm HST 1 BANK 1 HST 1 BIGFO 0.170020E+06 HST 1 CHANS 120 1 2214 7452672 2932 0 0 HST 1 EPHAS 0 0 0 0 0 0 0 0 0 Y HST 1 ICONR 0.709237 0.713647 0.000 0 0.5 0 0 HST 1 ICONS 0.709237 0.713647 0.000 0 0.5 0 0 HST 1 INAME NCCPAN HST 1 IRAD 3 HST 1 MAXRF 108 HST 1 NEXC 2 HST 1 NFOBS 1617 0 0 0 0 0 0 0 0 HST 1 NPHAS 1 0 0 0 0 0 0 0 0 HST 1 NREF 1636 1.0368 Y Y HST 1 R-FAC 1617 0.13685 4.125922E+06 HST 1 RPOWD 0.0214 0.0162 2214 5614.9 0.0000 0.0000 0 HST 1 TRNGE 3.01372 39.99516 HST 1 WREXP 0.0134 HST 1ABSCOR 0.000000E+00 0.000000E+00 N 0 HST 1BAKGD 1 1 Y 0 Y HST 1BAKGD1 0.212250E+04 HST 1CHI 0.0000 HST 1DETAZM 0.0000 HST 1DFUS 11 9 N HST 1DFUS 1 1 -0.12380E+04 0.7900 0.0500 YNNO O HST 1DFUS 2 1 -0.42219E+03 1.9100 0.0500 YNNSI O HST 1DFUS 3 1 0.33979E+02 3.3200 0.0500 YNNSI SI HST 1DFUS 4 1 -0.13992E+02 6.9100 0.0500 YNNSI SI HST 1DFUS 5 1 0.35242E+01 9.4100 0.0500 YNNSI SI HST 1DFUS 6 1 0.10108E+04 0.5100 0.0500 YNNO O HST 1DFUS 7 1 0.39007E+03 1.4200 0.0500 YNNSI O HST 1DFUS 8 1 0.22027E+03 2.2200 0.0500 YNNO O HST 1DFUS 9 1 -0.13626E+02 3.7200 0.0500 YNNSI SI HST 1DFUS10 1 0.64639E+01 6.6600 0.0500 YNNSI SI HST 1DFUS11 1 -0.23142E+02 8.1500 0.0500 YNNSI SI HST 1EXC 1 0.000 3.000 HST 1EXC 2 40.000 1000.000 HST 1EXMNMX 1.00000 1.00000 HST 1HSCALE 1.000000E+00 N 0 HST 1I HEAD SRM 640d Si 24 Jun 2016 0.25 div 0.02 Soller 1 mm cap HST 1I ITYP 0 1.0000 130.0000 1 HST 1MNREF 0 1.0368 HST 1ODMNMX 0.76474 1.25573 HST 1OMEGA 0.0000 Y HST 1PHI 0.0000 HST 1PRCF1 2 18 0.01 HST 1PRCF11 1.818400E+02 0.000000E+00 0.117200E+00 2.995000E+00 HST 1PRCF12 0.000000E+00 0.000000E+00 1.606000E+00 0.000000E+00 HST 1PRCF13 0.000000E+00 0.000000E+00 0.000000E+00 0.000000E+00 HST 1PRCF14 0.000000E+00 0.000000E+00 0.000000E+00 0.000000E+00 HST 1PRCF15 0.000000E+00 0.000000E+00 HST 1PRCF2 3 13 0.01 HST 1PRCF21 1.869000E+02 0.000000E+00 0.009000E+00 0.000000E+00 HST 1PRCF22 2.748000E+00 0.000000E+00 0.160000E-01 0.160000E-01 HST 1PRCF23 0.000000E+00 0.000000E+00 0.000000E+00 0.000000E+00 HST 1PRCF24 0.000000E+00 HST 1PRCF3 4 12 0.01 HST 1PRCF31 1.869000E+02 0.000000E+00 0.100000E+00 0.000000E+00 HST 1PRCF32 2.749000E+00 0.000000E+00 0.000700E+00 0.000000E+00 HST 1PRCF33 0.000000E+00 0.160000E-01 0.160000E-01 0.900000E+00 HST 1RSP 0.0258 0.0144 0.0150 0.0126 0.0173 0.0185 0.0148 0.0151 0.0176 HST 1RSPA 0.0304 0.0157 0.0156 0.0121 0.0202 0.0188 0.0139 0.0174 0.0187 HST 1RSPTT 3.01 7.11 11.22 15.33 19.44 23.55 27.66 31.77 35.88 40.00 HST 1RSPW 0.0353 0.0184 0.0190 0.0161 0.0220 0.0240 0.0188 0.0200 0.0220 HST 1RSPWA 0.0362 0.0215 0.0202 0.0153 0.0282 0.0244 0.0179 0.0267 0.0276 HST 1TRMNMX 1.00000 1.00000 HST 2 HNAM Bond distance restraints HST 2 FACTR 100.000 HST 2 NBNDS 12 HST 2 RMSD 12 0.0069 64.1178 HST 2BD0001 1 1 2 1 0 0 0 0 1.510 0.010 HST 2BD0002 1 4 5 1 0 0 0 0 1.510 0.010 HST 2BD0003 1 3 6 1 0 0 0 0 1.550 0.010 HST 2BD0004 1 3 17 1 0 0 0 0 1.420 0.030 HST 2BD0005 1 2 3 1 0 0 0 0 1.540 0.010 HST 2BD0006 1 3 4 1 0 0 0 0 1.540 0.010 HST 2BD0007 1 1 11 1 0 0 0 0 1.270 0.030 HST 2BD0008 1 1 12 1 0 0 0 0 1.270 0.030 HST 2BD0009 1 5 13 1 0 0 0 0 1.270 0.030 HST 2BD000A 1 5 14 1 0 0 0 0 1.270 0.030 HST 2BD000B 1 6 15 1 0 0 0 0 1.270 0.030 HST 2BD000C 1 6 16 1 0 0 0 0 1.270 0.030 HST 3 HNAM Bond angle restraints HST 3 FACTR 1.00000 HST 3 NANGS 17 HST 3 RMSD 17 11.1078 233.055 HST 3AN 1 1 3 109.00 3.00 0001 0002 0003 HST 3AN 2 1 3 109.00 3.00 0003 0004 0005 HST 3AN 3 1 3 109.00 3.00 0002 0003 0004 HST 3AN 4 1 3 109.00 3.00 0002 0003 0006 HST 3AN 5 1 3 109.00 3.00 0002 0003 0011 HST 3AN 6 1 3 109.00 3.00 0004 0003 0006 HST 3AN 7 1 3 109.00 3.00 0004 0003 0011 HST 3AN 8 1 3 109.00 3.00 0006 0003 0011 HST 3AN 9 1 3 120.00 3.00 000B 0001 000C HST 3AN 10 1 3 120.00 3.00 000B 0001 0002 HST 3AN 11 1 3 120.00 3.00 000C 0001 0002 HST 3AN 12 1 3 120.00 3.00 000D 0005 000E HST 3AN 13 1 3 120.00 3.00 000D 0005 0004 HST 3AN 14 1 3 120.00 3.00 000E 0005 0004 HST 3AN 15 1 3 120.00 3.00 000F 0006 0010 HST 3AN 16 1 3 120.00 3.00 000F 0006 0003 HST 3AN 17 1 3 120.00 3.00 0010 0006 0003 LNCN 1 1 1 2UISO 1.00001 3UISO 1.00001 4UISO 1.00001 7UISO 1.3000 LNCN 1 2 1 8UISO 1.30001 9UISO 1.30001 10UISO 1.3000 LNCN 2 1 1 1UISO 1.00001 5UISO 1.00001 6UISO 1.00001 11UISO 1.0000 LNCN 2 2 1 12UISO 1.00001 13UISO 1.00001 14UISO 1.00001 15UISO 1.0000 LNCN 2 3 1 16UISO 1.00001 17UISO 1.00001 18UISO 1.3000 LNCN 3 1 1 20UISO 1.00001 21UISO 1.0000 ZZZZZZZZZZZZ Last EXP file record

### dilithium potassium citrate (KADU1665_publ) Fractional atomic coordinates and isotropic or equivalent isotropic displacement parameters (Å^2^)

**Table:**

	*x*	*y*	*z*	*U*_iso_*/*U*_eq_	
C1	−0.155 (3)	0.066 (3)	0.3268 (18)	0.060 (3)*	
C2	−0.028 (3)	0.232 (3)	0.353 (2)	0.023 (5)*	
C3	0.187 (3)	0.251 (2)	0.2633 (18)	0.023 (5)*	
C4	0.246 (3)	0.429 (3)	0.3377 (18)	0.023 (5)*	
C5	0.435 (3)	0.528 (3)	0.268 (2)	0.060 (3)*	
C6	0.118 (4)	0.348 (3)	0.129 (2)	0.060 (3)*	
H7	−0.00317	0.21693	0.46524	0.030 (7)*	
H8	−0.12279	0.38491	0.33912	0.030 (7)*	
H9	0.27605	0.37435	0.44502	0.030 (7)*	
H10	0.10903	0.55430	0.35021	0.030 (7)*	
O11	−0.309 (2)	0.025 (2)	0.4174 (18)	0.060 (3)*	
O12	−0.107 (2)	−0.033 (2)	0.2162 (14)	0.060 (3)*	
O13	0.536 (3)	0.667 (3)	0.2988 (15)	0.060 (3)*	
O14	0.547 (3)	0.4306 (19)	0.1691 (15)	0.060 (3)*	
O15	0.006 (3)	0.520 (2)	0.1405 (14)	0.060 (3)*	
O16	0.208 (3)	0.215 (2)	0.0417 (16)	0.060 (3)*	
O17	0.327 (2)	0.073 (2)	0.2251 (17)	0.060 (3)*	
H18	0.47033	0.13035	0.22684	0.069 (3)*	
K19	0.7405 (10)	0.1861 (9)	−0.0297 (6)	0.046 (3)*	
Li20	0.743 (9)	0.746 (7)	0.163 (5)	0.05*	
Li21	−0.449 (8)	0.101 (7)	0.638 (5)	0.05*	

### dilithium potassium citrate (KADU1665_publ) Geometric parameters (Å, º)

**Table:**

C1—C2	1.5101 (16)	O15—K19^ii^	3.593 (15)
C1—O11	1.274 (5)	O15—K19^v^	2.784 (17)
C1—O12	1.273 (5)	O15—Li20^ii^	2.12 (5)
C2—C1	1.5101 (16)	O16—C3	2.18 (2)
C2—C3	1.5407 (16)	O16—C6	1.286 (5)
C3—C2	1.5407 (16)	O16—K19^ii^	3.251 (19)
C3—C4	1.5404 (16)	O16—K19	3.39 (2)
C3—C6	1.5507 (16)	O16—K19^iii^	2.664 (15)
C3—O17	1.433 (5)	O16—Li20^v^	1.99 (5)
C4—C3	1.5404 (16)	O17—C3	1.433 (5)
C4—C5	1.5099 (16)	O17—K19	3.488 (17)
C5—C4	1.5099 (16)	O17—K19^iii^	2.756 (16)
C5—O13	1.271 (5)	O17—Li21^vii^	1.95 (4)
C5—O14	1.270 (5)	K19—O12^viii^	3.015 (17)
C6—C3	1.5507 (16)	K19—O12^iii^	2.865 (12)
C6—O15	1.281 (5)	K19—O13^v^	3.473 (16)
C6—O16	1.286 (5)	K19—O14	2.650 (12)
O11—C1	1.274 (5)	K19—O14^v^	3.364 (16)
O11—Li21	2.26 (5)	K19—O15^viii^	3.593 (15)
O11—Li21^i^	2.02 (4)	K19—O15^v^	2.784 (17)
O12—C1	1.273 (5)	K19—O16	3.39 (2)
O12—K19^ii^	3.015 (17)	K19—O16^viii^	3.251 (19)
O12—K19^iii^	2.865 (12)	K19—O16^iii^	2.664 (15)
O12—Li20^iv^	1.99 (5)	K19—O17	3.488 (17)
O13—C5	1.271 (5)	K19—O17^iii^	2.756 (16)
O13—O14	2.06 (2)	Li20—O12^ix^	1.99 (5)
O13—K19^v^	3.473 (16)	Li20—O13	1.84 (5)
O13—Li20	1.84 (5)	Li20—O14	2.58 (6)
O13—Li21^vi^	1.69 (4)	Li20—O15^viii^	2.12 (5)
O14—C5	1.270 (5)	Li20—O16^v^	1.99 (5)
O14—O13	2.06 (2)	Li21—O11	2.26 (5)
O14—K19	2.650 (12)	Li21—O11^i^	2.02 (4)
O14—K19^v^	3.364 (16)	Li21—O13^vi^	1.69 (4)
O14—Li20	2.58 (6)	Li21—O17^vii^	1.95 (4)
O15—C6	1.281 (5)		
			
C2—C1—O11	119.6 (15)	K19^ii^—O16—Li20^v^	78.7 (18)
C2—C1—O12	120.5 (15)	K19^iii^—O16—Li20^v^	93.8 (16)
O11—C1—O12	119.9 (17)	C3—O17—K19^iii^	122.5 (11)
C1—C2—C3	120.0 (15)	C3—O17—Li21^vii^	122 (2)
C2—C3—C4	97.8 (12)	K19^iii^—O17—Li21^vii^	104.8 (14)
C2—C3—C6	100.9 (16)	O12^viii^—K19—O12^iii^	93.1 (4)
C2—C3—O17	119.5 (14)	O12^viii^—K19—O14	80.2 (4)
C4—C3—C6	104.0 (16)	O12^viii^—K19—O15^v^	112.5 (5)
C4—C3—O17	124.1 (16)	O12^viii^—K19—O16^viii^	58.1 (4)
C6—C3—O17	107.4 (13)	O12^viii^—K19—O16^iii^	64.6 (5)
C3—C4—C5	116.9 (15)	O12^viii^—K19—O17^iii^	112.6 (6)
C4—C5—O13	135.4 (16)	O12^iii^—K19—O14	151.6 (4)
C4—C5—O14	114.8 (16)	O12^iii^—K19—O15^v^	66.0 (4)
O13—C5—O14	108.1 (16)	O12^iii^—K19—O16^viii^	59.4 (4)
C3—C6—O15	116.7 (14)	O12^iii^—K19—O16^iii^	66.9 (5)
C3—C6—O16	100.0 (13)	O12^iii^—K19—O17^iii^	64.5 (5)
O15—C6—O16	143.3 (19)	O14—K19—O15^v^	90.9 (4)
C1—O11—Li21	139 (2)	O14—K19—O16^viii^	94.4 (5)
C1—O11—Li21^i^	121 (3)	O14—K19—O16^iii^	131.2 (5)
Li21—O11—Li21^i^	99 (2)	O14—K19—O17^iii^	143.4 (5)
C1—O12—K19^ii^	113.4 (18)	O15^v^—K19—O16^viii^	56.1 (4)
C1—O12—K19^iii^	139.2 (14)	O15^v^—K19—O16^iii^	132.5 (6)
C1—O12—Li20^iv^	126.4 (19)	O15^v^—K19—O17^iii^	112.9 (5)
K19^ii^—O12—K19^iii^	86.9 (4)	O16^viii^—K19—O16^iii^	94.3 (5)
K19^ii^—O12—Li20^iv^	83.9 (18)	O16^viii^—K19—O17^iii^	121.7 (5)
K19^iii^—O12—Li20^iv^	89.2 (16)	O16^iii^—K19—O17^iii^	48.0 (5)
C5—O13—Li20	116 (2)	O12^ix^—Li20—O13	114 (3)
C5—O13—Li21^vi^	131 (3)	O12^ix^—Li20—O15^viii^	97 (3)
Li20—O13—Li21^vi^	97 (3)	O12^ix^—Li20—O16^v^	100 (3)
C5—O14—K19	171.2 (13)	O13—Li20—O15^viii^	110 (2)
C6—O15—K19^v^	108.2 (17)	O13—Li20—O16^v^	138 (3)
C6—O15—Li20^ii^	162 (2)	O15^viii^—Li20—O16^v^	88 (2)
K19^v^—O15—Li20^ii^	88.7 (14)	O11—Li21—O13^vi^	127 (3)
C6—O16—K19^ii^	85.9 (16)	O11—Li21—O17^vii^	114 (2)
C6—O16—K19^iii^	137.0 (15)	O11^vii^—Li21—O13^vi^	110 (3)
C6—O16—Li20^v^	125 (2)	O11^i^—Li21—O17^vii^	109 (2)
K19^ii^—O16—K19^iii^	85.7 (5)	O13^vi^—Li21—O17^vii^	111 (3)

Symmetry codes: (i) −*x*−1, −*y*, −*z*+1; (ii) *x*−1, *y*, *z*; (iii) −*x*+1, −*y*, −*z*; (iv) *x*−1, *y*−1, *z*; (v) −*x*+1, −*y*+1, −*z*; (vi) −*x*, −*y*+1, −*z*+1; (vii) −*x*, −*y*, −*z*+1; (viii) *x*+1, *y*, *z*; (ix) *x*+1, *y*+1, *z*.

### (KADU1665_DFT) Crystal data

**Table:**

[Li_2_K(C_6_H_5_O_7_)]	*c* = 9.8171 Å
*M**_r_* = 242.08	α = 87.6370°
Triclinic, *P*1	β = 80.6060°
Hall symbol: -P 1	γ = 83.1090°
*a* = 6.4842 Å	*V* = 416.59 Å^3^
*b* = 6.6833 Å	*Z* = 2

### (KADU1665_DFT) Data collection

**Table:**

*h* = →	*l* = →
*k* = →	

### (KADU1665_DFT) Fractional atomic coordinates and isotropic or equivalent isotropic displacement parameters (Å^2^)

**Table:**

	*x*	*y*	*z*	*U*_iso_*/*U*_eq_	
C1	−0.14556	0.04948	0.32403	0.05980*	
C2	−0.01819	0.21103	0.36266	0.02280*	
C3	0.19131	0.25426	0.27686	0.02280*	
C4	0.28017	0.41395	0.35448	0.02280*	
C5	0.48732	0.47534	0.27699	0.05980*	
C6	0.16663	0.34606	0.13181	0.05980*	
H7	0.00957	0.17532	0.46825	0.02960*	
H8	−0.12413	0.35150	0.36689	0.02960*	
H9	0.30601	0.35306	0.45644	0.02960*	
H10	0.16579	0.54770	0.36996	0.02960*	
O11	−0.30283	0.02010	0.41651	0.05980*	
O12	−0.09949	−0.04238	0.21200	0.05980*	
O13	0.54051	0.64716	0.29975	0.05980*	
O14	0.59879	0.35337	0.19339	0.05980*	
O15	0.07044	0.52106	0.13348	0.05980*	
O16	0.25196	0.24714	0.02620	0.05980*	
O17	0.33736	0.07475	0.26331	0.05980*	
H18	0.47033	0.13035	0.22684	0.06880*	
K19	0.72704	0.16395	−0.04174	0.04610*	
Li20	0.80495	0.68748	0.16168	0.05000*	
Li21	−0.44493	0.11043	0.59780	0.05000*	

### (KADU1665_DFT) Bond lengths (Å)

**Table:**

C1—C2	1.530	C4—C5	1.525
C1—O11	1.278	C4—H9	1.096
C1—O12	1.257	C4—H10	1.090
C2—C3	1.529	C5—O13	1.276
C2—H7	1.093	C5—O14	1.257
C2—H8	1.093	C6—O15	1.257
C3—C4	1.552	C6—O16	1.265
C3—C6	1.552	O17—H18	0.987
C3—O17	1.433		

### (KADU1665_DFT) Hydrogen-bond geometry (Å, º)

**Table:**

*D*—H···*A*	*D*—H	H···*A*	*D*···*A*	*D*—H···*A*
O17—H18···O14	0.987	1.786	2.662	145.8
C4—H10···O15	1.090	2.516	2.770	91.6
